# Ultrasound-guided retro-neural transforaminal injection with fluoroscopic confirmation for zoster-associated pain: a retrospective propensity score-matched analysis

**DOI:** 10.3389/fpain.2026.1768584

**Published:** 2026-05-20

**Authors:** Xiuhua Li, Shuyue Zheng

**Affiliations:** 1 Department of Anesthesiology, Beijing Tongren Hospital, Capital Medical University, Beijing, China; 2 Department of Pain Management, Capital Medical University Affiliated Beijing Shijitan Hospital, Beijing, China

**Keywords:** post-herpetic neuralgia, retro-neural, transforaminal injection, ultrasound, zoster-associated pain

## Abstract

**Objectives:**

This study aimed to evaluate the efficacy and safety of an ultrasound (US)-guided lumbar transforaminal steroid injection (TFI) using the retro-neural approach for relieving acute zoster-associated pain (ZAP) in the lower extremities.

**Methods:**

A total of 72 patients who underwent US-guided retro-neural TFI were retrospectively stratified as the US cohort. Furthermore, 1:1 propensity score matching was performed to select controls who underwent fluoroscopy (FL)-guided procedures. The primary endpoint was visual analog scale (VAS) pain scores at 1 month after the procedure. Secondary outcomes included sensory blockade, procedure duration, radiation exposure, adverse events, rescue analgesic use, post-herpetic neuralgia (PHN) incidence, and European Quality of Life Five-Dimension questionnaire (EQ-5D) score.

**Results:**

At 1 month, the US cohort reported a mean VAS score of 33.52 ± 14.30 mm with a mean difference of 1.04 mm compared to the FL cohort, indicating non-inferiority as the upper limit of the 95% confidence interval of 4.36 mm fell within the non-inferior margin of 10 mm. PHN occurred in 16.7% and 13.9% of cases in the US and FL cohorts at 3 months after rash onset (*p* = 0.796), respectively. Both cohorts showed similar improvements in 3-month VAS scores (*p* = 0.310), rescue analgesics (all *p* > 0.05), and EQ-5D scores (all *p* > 0.05). Compared to the FL cohort, the US approach was associated with a wider anesthetic dermatome [3.25 (IQR: 2.75, 3.75) vs. 3 (IQR: 2.5, 3.5) levels for 3 mL of therapeutic injectate, *p* = 0.031], fewer needle insertions [1 (IQR: 0, 2) vs. 2.5 (IQR: 1.5, 3.5), *p* = 0.005], shorter procedure duration (14.09 ± 3.58 vs. 19.70 ± 4.84 min, *p* < 0.001), and less radiation exposure (1,226.89 ± 377.28 vs. 5,607.50 ± 1,391.38 µGy·m^2^, *p* < 0.001). No serious adverse events were observed.

**Conclusions:**

US-assisted lumbar retro-neural RFI was statistically non-inferior to conventional procedures under FL guidance for treating ZAP in the lower extremities. It provided the benefits of fewer needle attempts, a shorter procedure duration, and decreased radiation exposure, supporting its use in clinical routine practice.

## Introduction

Herpes zoster (HZ) is caused by the reactivation of dormant varicella-zoster virus (VZV) in dorsal root ganglion cells after a primary infection due to immunocompromise (1). The incidence of HZ in China was 3–5 per 1,000 person-years (PY) across all age groups, increasing to 11.07 and 13.63 per 1,000 PYs for individuals aged over 60 and 80 years (2). Intra-nerve spread of VZV after reactivation contributes to vesicular rashes and acute zoster-associated pain (ZAP) in the majority of cases (3). Although the eruption of rashes is self-limiting, a considerable proportion of HZ cases subsequently develop debilitating post-herpetic neuralgia (PHN) after the skin lesions have healed over several weeks (4). The chronic neuropathic pain is very challenging to treat, significantly reducing health-related quality of life (QoL) and imposing the largest healthcare burdens related to HZ (5). As a result, early recognition and treatment of ZAP should be prioritized to prevent its progression to PHN (6). Fluoroscopy (FL)-guided transforaminal steroid injection (TFI) is effective for the treatment of ZAP within 12 weeks of HZ onset due to its superior accessibility to the dorsal root ganglion and has been recommended by the Neuropathic Pain Special Interest Group for patients who do not respond to conservative analgesics (7, 8). Recent studies show that ultrasound (US) imaging is a reliable alternative to overcome the radiological disadvantages associated with guiding lumbar epidural injections via the transforaminal approach in patients with lumbar radicular pain (9). However, there is limited evidence regarding its utilization in managing herpes zoster-related pain (10).

This study employed propensity score matching (PSM) to control for confounding variables and aimed to evaluate the efficacy and safety of US-guided lumbar TFI using the retro-neural approach and controlled by FL imaging for the treatment of herpes zoster radiculopathy.

## Methods

This retrospective study was approved by the institutional Human Ethics Committees in accordance with the Declaration of Helsinki principles and following the Strengthening the Reporting of Observational Studies in Epidemiology (STROBE) guidelines (11). The need for written informed consent was waived because all data were collected from the medical records.

**Figure 1:**
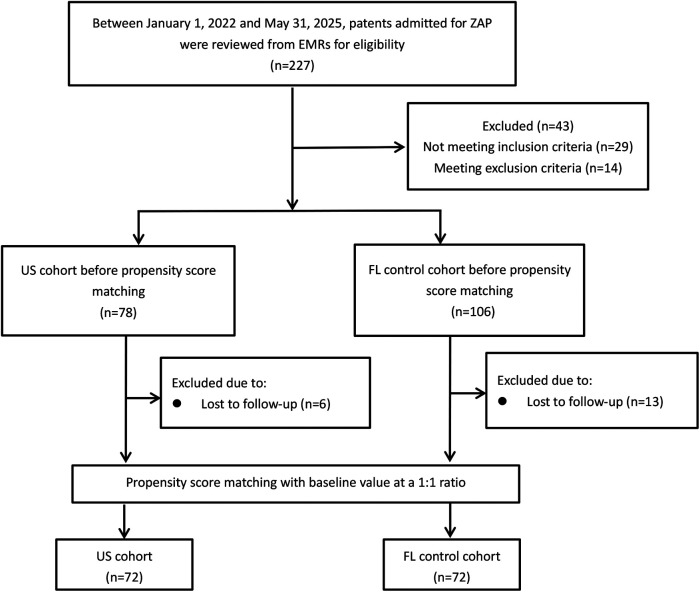
Flowchart of the study cohorts. ZAP, acute zoster-associated pain; US, ultrasound; FL, fluoroscopy.

Between 1 January 2022 and 31 May 2025, patients admitted for the treatment of HZ in the lower extremities were reviewed for eligibility using the electronic medical records (Figure 1). Inclusion criteria were as follows: (1) a clinical diagnosis of HZ in a unilateral lower extremity; (2) involvement of two or more dermatomes; (3) intensity of ZAP ≥40 mm on the visual analog scale (VAS); (4) disease duration within 4 weeks after rash onset; (5) pain relief <50% after a 7-day course of antiviral therapy with valaciclovir; and (6) ≥50 years of age. Patients with immune deficiency, coagulation disorders, severe hepatic or renal dysfunction, severe heart failure, psychiatric illness, severe cognitive impairment, an allergy to contrasts, systemic use of analgesics, pregnancy or lactation, a high iliac crest, conversion to other procedures, and incomplete data were excluded.

During outpatient consultations, patients were provided with information about TFI based on guidelines, using both the US and FL techniques. Following this, patients made the treatment decision that most aligned with their values and preferences. Both the US and FL procedures were performed in an outpatient operating room by a team of four pain specialists with expertise in minimally invasive treatments for HZ. The procedures were randomly assigned to each specialist according to the patients’ admission sequence. A PSM approach was performed to control for selection bias due to the non-random cohort assignment. Propensity scores were calculated using logistic regression and the nearest neighbor technique with a predefined caliper of 0.1 based on the following variables: (1) age; (2) gender; (3) affected side; (4) duration after rash onset and duration from antiviral therapeutic failure; (5) rash severity; (6) hemorrhagic lesions; (7) baseline VAS pain scores; (8) number of involved dermatomes; (9) type of analgesics; and (10) comorbidities. After 1:1 matching, each cohort contained 72 patients.

### US-guided lumbar retro-neural TFI procedure

**Figure 2:**
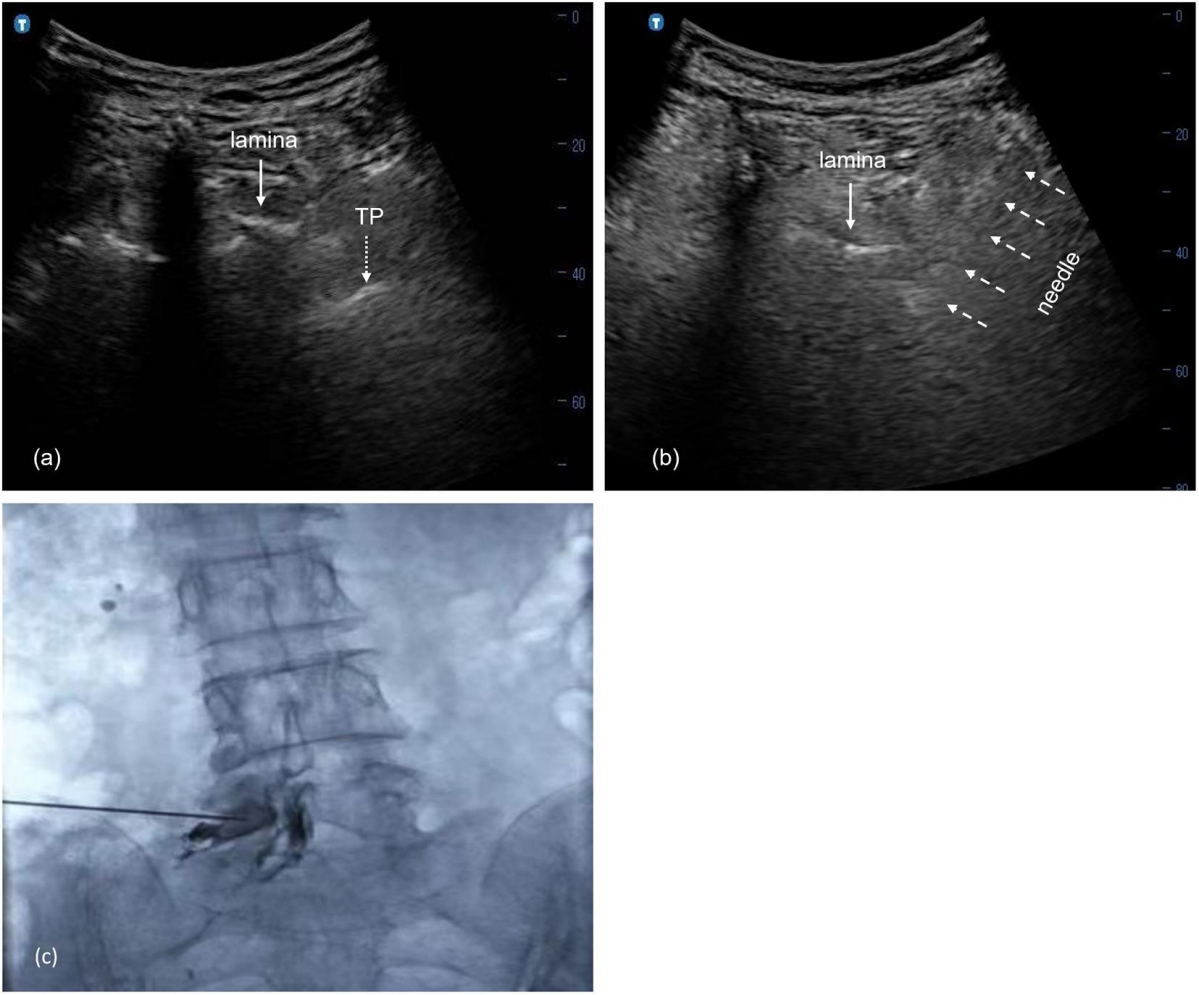
**(a)** Transverse view of US image at L5 intervertebral foramen; **(b)** selective nerve root (L5) block using the transforaminal approach under US guidance; the needle (dotted arrow) is advanced into the foramen using the in-plane technique; **(c)** contrast medium disperses into the L5 epidural and foraminal spaces in the fluoroscopic AP view. US, ultrasound; FL, fluoroscopy; AP, anterior–posterior; TP, transverse.

A low-frequency (2–5 MHz) transducer was positioned at 2–4 cm lateral to the midline of the lumbar spine to identify the transverse processes (TPs) using a sagittal approach (Figure 2a). It was then rotated 90° to visualize the targeted TP in the short-axis view. The armpit of the lumbar lamina appeared as a clear hyper-echoic line when the transducer was slightly moved caudally until the TP disappeared. The retro-neural aspect of the intervertebral foramen was easily detected by sliding along the bony barrier of the armpit within a distance of 3–5 mm. A careful scan for nearby vessels around the projected needle trajectory was performed using the color Doppler mode. After local anesthesia with 0.5% lidocaine, a 22-gauge needle was slowly advanced in a lateral-to-medial direction using the in-plane technique under real-time guidance (Figure 2b). An anteroposterior (AP) fluoroscopic scan was performed in all US cases to verify the position of the needle tip, which was not allowed to exceed the 6 o’clock position of the lumbar vertebral pedicle. After negative aspiration, a total of 1 mL of contrast was injected to detect the dispersion pattern (Figure 2c). Subsequently, a total volume of 1× the number of involved nerves mL of a therapeutic mixture [lidocaine (2 mg/mL) + dexamethasone (1.5 mg/mL)] was injected slowly under real-time US guidance based on previous evidence (12).

### FL-guided retro-neural TFI procedure

The C-arm was tilted to be parallel to the endplates of the targeted lumbar level, and then rotated 20°–30° to visualize the Scotty dog sign, which corresponds to the vertebral pedicle. The same puncture needle used in the US cohort was advanced to the 6 o’clock position of the pedicle on the ipsilateral oblique view using the tubular vision technique. After verifying that the needle tip was at the anterosuperior aspect of the intervertebral foramen with negative aspiration, the same volume of contrast as the US cohort was injected to confirm epidural spread on both AP and lateral views. Finally, the same injectate as the US cohort was administered under FL guidance.

### Rescue analgesics

Rescue analgesics were permitted upon request after the procedure. If a patient reported a VAS pain score <40 mm, celecoxib, at a dosage of 200 mg per tablet, up to two times daily, was administered. For patients with a VAS pain score ≥40 mm, tramadol hydrochloride, at a dosage of 100 mg per tablet, up to twice daily, was provided following the World Health Organization’s analgesic ladder (13).

### Outcome measurement

Demographic and clinical data were extracted from the electronic medical records. Follow-up data were retrieved from outpatient documents at 1 and 3 months after the TFI procedure.

Sensory blockade to cold was assessed using an alcohol-based cotton ball at 5 min after the injection. Motor blockade was measured using the Bromage score, which is graded from 0 (no motor block) to 3 (complete motor block) (14). Needle attempts were defined as the number of times the needle was redirected before reaching the target position. Procedure duration was predefined as the interval between the start of the initial imaging and the end of the injection. Radiation doses were determined as the dose area product generated by the C-arm during the procedure. Pain severity was measured using the VAS with a 100 mm line ranging from 0 mm (no pain) to 100 mm (worst pain) (15). PHN was defined as pain persisting with a VAS pain score >0 for more than 90 days after rash onset (16). Health-related QoL was assessed by the European Quality of Life 5-Dimension questionnaire (EQ-5D), which measures five dimensions including mobility, self-care, usual activities, pain/discomfort, and anxiety/depression. Responses for each dimension were rated as no, some, or extreme problems (17).

The primary endpoint was the mean VAS pain score at 1 month after procedure, following the recommendations of the Herpes Zoster Clinical Trial Consensus Group (18, 19). Secondary outcomes included sensory and motor blockade, procedure duration, radiation exposure, PHN incidence, rescue analgesic use, EQ-5D score, and adverse events.

### Sample size calculation

The sample size was calculated using PASS statistical software, version 19.0 (NCSS, LLC, Kaysville, Utah, USA). Based on prior evidence, patients with a reported HZ-related pain intensity of a mean VAS score of 32 mm should receive epidural injections at 1 month after the intervention (8). Considering the benefits of US guidance, the new treatment would be adopted if the VAS pain scores at 1 month in this cohort were slightly worse than those in the FL approach cohort. A non-inferiority margin of 10 mm was assumed because a decrease of at least 30 mm was aligned with the guideline for the minimal clinically important difference associated with effective pain management in patients with HZ, while the actual difference ranged from −5 to 5 mm (20). To achieve a power of 0.8 and a Type I error of 0.025, the required sample size was 64 patients per cohort. Accounting for a 20% data loss, the final sample size was calculated as 80 cases per cohort.

### Statistical analysis

Statistical analysis was performed using SPSS software version 30.0 (SPSS Inc., Chicago, IL, USA). Statistical significance was set at *p* < 0.05. The Kolmogorov–Smirnov *Z* test was used to test for normality. Normal data, skewed quantitative data, and categorical data were described as mean ± standard deviation (SD), median (interquartile range, IQR), and frequency/percentage, respectively. Comparisons between cohorts were performed using Student’s *t* test, the Mann–Whitney U test, and Fisher's exact test. Repeated measures analysis of variance (rm-ANOVA) was employed to compare the normal data over time. The Bonferroni correction was used for the *post-hoc* comparison (adjusted *α* of 0.05/3 = 0.017).

## Results

Figure 1 shows the flowchart of the study cohorts. There were no significant differences in the patients’ demographic and clinical characteristics between the two cohorts at baseline (Table 1).

**Table 1:** Baseline characteristics of the patients in the two cohorts.

Variable	Before PSM				SMD	After PSM				SMD
	US cohort (*n* = 95)	FL cohort (*n* = 153)	*t*/χ^2^ value	*p*		US cohort (*N* = 72)	FL cohort (*N* = 72)	*t*/χ^2^ value	*p*	
Age ± SD (years）	65.82 ± 3.54	67.12 ± 3.28	−2.943	0.004	−0.381	66.82 ± 3.59	67.06 ± 3.76	−0.392	0.696	−0.065
Gender, *n* (%)			0.083	0.794				0.028	0.868	
Female	51 (53.7%)	85 (55.6%)			−0.038	37 (51.4%)	38 (52.8%)			−0.028
Male	44 (46.3%)	68 (44.4%)			0.038	35 (48.6%)	34 (47.2%)			0.028
Affected side, *n* (%)			0.275	0.693				0.250	0.739	
Left	56 (58.9%)	85 (55.6%)			0.023	39 (54.2%)	36 (50.0%)			0.084
Right	39 (41.1%)	68 (44.4%)			−0.023	33 (45.8%)	36 (50.0%)			−0.084
Duration after rash onset ± SD (days)	21.03 ± 2.78	20.89 ± 2.27	0.433	0.666	0.062	20.02 ± 2.35	19.98 ± 2.21	0.107	0.915	0.018
Duration from antiviral therapeutic failure ± SD (days)	13.72 ± 3.14	12.95 ± 3.37	1.418	0.158	0.236	13.05 ± 3.28	12.98 ± 3.17	0.130	0.897	0.022
VAS pain score ± SD	76.78 ± 11.93	75.36 ± 10.75	0.969	0.333	0.125	77.86 ± 12.99	78.40 ± 13.94	−0.755	0.450	−0.040
Affected lumbosacral dermatomes, *n* (%)			0.895	0.387				0.138	0.853	
Two levels	72 (84.7%)	122 (79.7%)			0.131	53 (73.6%)	51 (70.8%)			0.063
Three levels	13 (15.3%)	31 (20.3%)			−0.131	19 (26.4%)	21 (29.2%)			−0.063
Rash severity, *n* (%)			0.461	0.547				0.161	0.841	
Number of lesions < 50	69 (72.6%)	117 (76.5%)			−0.090	55 (76.4%)	57 (79.2%)			−0.067
Number of lesions ≥ 50	26 (27.4%)	36 (23.5%)			0.090	17 (23.6%)	15 (20.8%)			0.067
Hemorrhagic lesion, *n* (%)			5.930	0.015				0.177	0.834	
No	80 (84.2%)	108 (70.6%)			0.329	59 (81.9%)	57 (79.2%)			0.068
Yes	15 (15.8%)	45 (29.4%)			−0.329	13 (18.1%)	15 (20.8%)			−0.068
History of analgesics, *n* (%)			27.567	<0.001				0.450	0.799	
None	34 (35.8%)	26 (17.0%)			0.499	23 (32.4%)	21 (29.7%)			0.058
NSAIDs	51 (53.7%)	66 (43.1%)			0.231	31 (44.0%)	34 (47.2%)			−0.064
Anti-epileptic or weak opioid	10 (10.5%)	61 (39.9%)			−0.719	18 (23.6%)	17 (23.1%)			0.012
Comorbidities, *n* (%)										
Hypertension	30 (31.6%)	27 (17.6%)	6.427	0.013	0.329	22 (30.6%)	20 (27.8%)	0.134	0.855	0.062
Coronary heart disease	24 (25.3%)	20 (13.1%)	1.388	0.017	0.314	14 (19.4%)	16 (22.2%)	0.168	0.838	−0.069
Diabetes mellitus	18 (18.9%)	19 (12.6%)	1.849	0.201	0.173	13 (18.1%)	11 (15.3%)	0.200	0.823	0.075

FL, fluoroscopy; US, ultrasound; SD, standard deviation; NSAIDs, non-steroidal anti-inflammatory drugs; PSM, propensity score matching; SMD, standardized mean difference.

**Figure 3:**
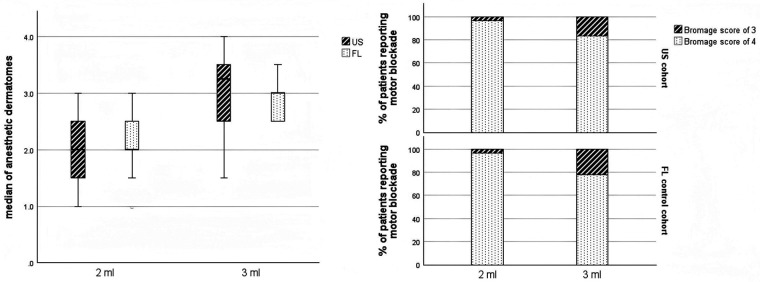
Sensory and motor blockades after TFI. The median number of anesthetic dermatomes is significantly higher among patients administered 3 mL of therapeutic injectate in the US cohort than the FL cohort. A higher proportion of patients presented with partial motor blockades with different volumes of therapeutic injectate in the FL cohort compared to the US cohort; however, the difference did not reach statistical significance. TFI, transforaminal epidural injection; FL, fluoroscopy; US, ultrasound.

The median number of anesthetic dermatomes in the US and FL cohorts was 2 (1, 3) and 2 (1.5, 2.5), respectively, in patients administered 2 mL of therapeutic injectate; however, the difference did not reach statistical significance (*p* = 0.492). For patients receiving a 3-mL injection, a median of 3.25 (2.75, 3.75) with sensory blockade was observed in the US cohort, which was significantly higher than that in the FL control cohort [3 (2.5, 3.5)](*p* = 0.031). Furthermore, the percentage of patients presenting with a partial motor blockade with a Bromage score of 3 was slightly lower in the US cohort compared to the FL controls after injecting 2 mL (4.2% vs. 6.9%, *p* = 0.791) or 3 mL (16.7% vs. 20.8%, *p* = 0.67) of therapeutic mixture (Figure 3). The median number of needle attempts before FL confirmation was 1 (IQR: 0, 2) (range: 1, 3) in the US cohort, which was lower than the FL cohort [2.5 (IQR: 1.5, 3.5) (range: 1, 4)] (*p* = 0.05). The mean procedure duration was 14.09 ± 3.58 min in the US cohort, which was shorter than that of the FL control cohort (19.70 ± 4.84) (*p* < 0.001). The mean number of fluoroscopic shots per procedure was 2.13 ± 1.51 in the US cohort, which was significantly less than in the FL cohort (5.81 ± 1.41) (*p* < 0.001). The patients who underwent TFI using ultrasound guidance were exposed to significantly less radiation, receiving nearly one-fifth the dose compared to those who had undergone the traditional fluoroscopy-guided procedure (1,226.89 ± 377.28 vs. 5,607.50 ± 1,391.38 µGy·m^2^, *p* < 0.001). Additionally, the average number of fluoroscopic images taken was 2.13 ± 0.92 in the ultrasound cohort, which was lower than the 4.36 ± 1.28 images in the fluoroscopy cohort (*p* < 0.001). With regard to adverse events, intravascular puncture was observed in 4.3% and 9.7% of the cases in the US and FL cohorts, respectively (*p* = 0.326). No serious side effects, such as spinal cord injury, total spinal anesthesia, epidural hematoma, local anesthetic intoxication, or infection, occurred in either cohort.

As shown in Table 2, the VAS pain scores in both cohorts were significantly reduced at 1 month after the injection compared to their baseline values, with a mean difference (MD) of 1.04 mm (95%CI: −2.28, 4.36). Non-inferiority was met because the 95% CI fell within the non-inferior margin of 10. According to the rm-ANOVA analysis, the mean VAS scores at the 3-month follow-up were comparable between the two cohorts. Moreover, no significant differences were observed between the two cohorts regarding the percentage of patients who reported complete resolution of ZAP at both 1 and 3 months after the procedure. As a result, the presence of PHN at 3 months after the onset of rash was 16.7% and 13.9% in the US and FL cohorts, respectively (*p* = 0.817). Additionally, similar use of rescue analgesics was also observed between the two cohorts across all timepoints, as shown in Table 3 (all *p* > 0.05).

**Table 2:** VAS score for pain severity and proportion of patients without HZ-related pain over time.

Outcome		US cohort (*n* = 72)	FL control cohort (*n* = 72)	Mean difference (95%CI)	*t*/χ^2^ value	*p*
VAS score, mm (mean ± SD)	1 month	33.52 ± 14.30	32.48 ± 12.27	1.04 (−2.28, 4.36)	0.617	0.538
	3 months	18.76 ± 12.53	17.03 ± 13.76	1.73 (−1.62, 5.07)	1.017	0.310
Complete remission, *n* (%)	1 month	32 (44.4%)	38 (52.8%)	−8.3% (−24.6%, 10.9%)	1.001	0.405
	3 months	53 (73.6%)	57 (79.2%)	−5.6% (−19.4%, 8.3%)	0.616	0.557

ZAP, acute zoster-associated pain; VAS, visual analog scale; FL, fluoroscopy; US, ultrasound; SD, standard deviation; CI, confidence interval.

**Table 3:** Comparison of rescue analgesic use between the two cohorts over time.

Outcome			US cohort (*n* = 72)	FL control cohort (*n* = 72)	*t*/χ^2^ value	*p*
Patients administered rescue analgesics, *n* (%)	Celecoxib	1 month	15 (20.8%)	18 (25.0%)	0.354	0.692
		3 months	4 (5.6%)	7 (9.7%)	0.886	0.532
	Tramadol hydrochloride	1 month	13 (18.1%)	10 (13.9%)	0.466	0.650
		3 months	8 (11.1%)	9 (12.5%)	0.067	0.796
Mean daily dose, mg (mean ± SD)	Celecoxib	1 month	293.33 ± 103.28	288.89 ± 102.26	0.124	0.902
		3 months	300.00 ± 115.47	314.29 ± 106.90	−0.208	0.840
	Tramadol hydrochloride	1 month	160.00 ± 45.95	138.46 ± 71.16	0.831	0.415
		3 months	161.11 ± 41.67	156.25 ± 41.73	0.240	0.814

US, ultrasound; FL, fluoroscopy; SD, standard deviation.

**Figure 4:**
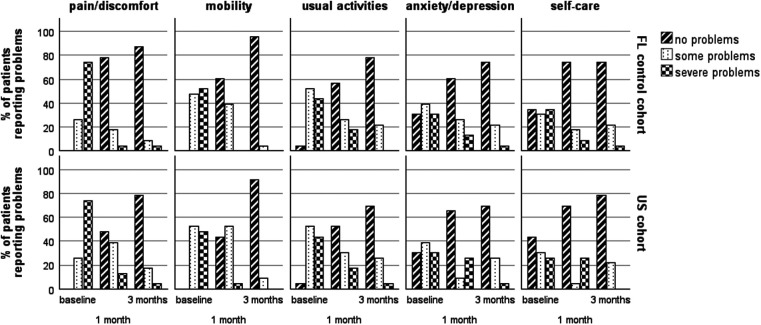
Theproportion of patients who reported problems in the five domains of the EQ-5D-3L over time. There were no significant differences between the two cohorts. EQ-5D-3L, the 3L version of the Euro-QoL five-dimensional questionnaire.

In terms of pain/discomfort, mobility, usual activities, anxiety/depression, and self-care, 72.3% and 73.9%, 78.3% and 89.1%, 52.2% and 56.5%, 65.2% and 60.8%, 69.6% and 73.9% of the patients in the US and FL cohorts, respectively, reported no problems at 1 month following the TFI procedure; however, the differences did not reach statistical significance (all *p* > 0.05). Similar trends were observed between the two cohorts at 3 months after the procedure (83.6% vs. 86.9%, 91.3% vs. 95.6%, 89.5% vs. 78.2%, 69.6% vs. 73.9%, and 78.2% vs. 73.9%, all *p* > 0.05) (Figure 4).

## Discussion

To our knowledge, this study is the first to investigate the early use of US-assisted lumbar TFI via the retro-neural approach in managing zoster-associated radiculopathy. Our findings showed that the decrease in VAS pain scores up to 1 month after injection did not significantly differ between the US-guided and standard FL methods, although the US cohort exhibited a shorter procedure duration and reduced radiation exposure.

Except for elderly and immunocompromised individuals, the increased risk of developing chronic PHN correlated with the severity of ZAP symptoms (21). In a meta-analysis, standard antiviral medications did not significantly prevent PHN, with a risk ratio (RR) of 1.05 for PHN events at 6 months after rash onset between the antiviral group and placebo (*p* = 0.62 ) (22). In contrast, a RR value of 0.45 indicated that a local anesthetic and steroid injection combination resulted in a 55% reduced risk of PHN at 3 months after rash onset (23). The beneficial effects include inhibiting the transmission of nociceptive afferent signals or suppressing the neural inflammatory reaction, therefore preventing peripheral and central sensitization to avoid PHN (24). TFI is a widely used technique for the treatment of lumbar radiculopathy because it allows better access to deliver therapeutic drugs to the involved dorsal root ganglion and nerve root (25). A prior study reported that a transforaminal epidural injection during acute HZ significantly reduced pain intensity after 1 and 3 months, with VAS pain scores lowered to 20 mm and 10 mm, respectively (26). Our data from the FL cohort demonstrated that the average VAS score was reduced to 32.48 mm and 17.03 mm at 1 and 3 months, respectively, with 52.8% and 79.2% of patients experiencing resolution of ZAP following a single lumbar epidural injection at these timepoints, respectively. These findings are consistent with the results of a randomized controlled trial published in The Lancet, which assigned 598 patients with ZAP to receive antiviral treatment with one additional epidural injection of 80 mg methylprednisolone acetate and 10 mg bupivacaine (8).

In contrast to conventional FL guidance, US imaging has been proven to be accurate in identifying the lumbar intervertebral foramen and assisting in performing transforaminal injections (27). In this study, the needle was inserted towards the retro-neural aspect of the neural foramen using the bony structure of the lumbar armpit as a hyper-echoic landmark on sonographic scanning, enabling the injectate to spread to the anterior epidural spaces and the concurrent dorsal space (27). The US cohort exhibited a narrower sensory blockade compared to the FL cohort after an injection of 3 mL of injectate, which may be due to a larger drug concentration reaching the ventral epidural space (28). Nevertheless, a ventral spreading pattern does not guarantee better clinical efficacy in relieving pain when performing a TFI procedure (29). In line with previous evidence, the severity of HZ-related pain significantly decreased over time in the US cohort. Regarding the primary endpoint, the mean VAS score at 1 month after US-guided TFI was non-inferior to that of the FL control cohort (*p* = 0.538). Additionally, our findings showed a significant decrease in the occurrence of PHN at 3 months after rash onset in both cohorts, which was similar to the results in a previous systematic review and meta-analysis that suggested that FL-guided epidural injections can effectively block invasive afferent nerves to manage ZAP (30).

With regard to QoL, in a previous study, the brief pain inventory scores for the dimensions of general activity, mood, walking ability, normal work, relationships with others, sleep, and enjoyment of life decreased significantly from baseline to 2 weeks and 3 months after epidural injection in patients with HZ, which confirmed the injection’s effect on health-related QoL (31). Consistent with this previous study, the EQ-5D-3L scores in both cohorts showed significant improvements after the intervention. However, no significant differences were observed between the two cohorts at any timepoint, suggesting similar improvement trends in the domains of pain, usual activities, mobility, anxiety, and self-care over time.

Recent evidence has indicated that the sub-pedicular approach is routinely used in lumbar TFI under FL guidance. However, this method places the puncture needle at the superoanterior aspect of the foramen, which leads to a higher risk of an inadvertent intravascular injection, occurring in 19% to 23% of cases. The increased risk was due to the presence of a radicular artery in 96.2% of cases that used this approach (32). Conversely, since only 2.6% of radicular arteries are located in the superoposterior aspect of the lumbar foramen, performing TFI via the retro-neural approach under US guidance was associated with a lower risk of an intravascular injection (27). According to our results, unintentional intravascular punctures were less frequent in the US cohort, indicating the safety of the US approach. US imaging not only identified nearby vessels using color Doppler to minimize adverse events but also facilitated needle insertion under real-time guidance to shorten the procedure duration and reduce radiation exposure. A prospective randomized clinical trial was conducted to compare US-guided vs. FL-controlled lumber TFI, which found that the operation duration in the US group (518 ± 103 s) was shorter than in the FL group (929 ± 228 s) (*P* < 0.05), leading to a lower radiation dose in the US group than the FL group (33).

This study had several limitations. First, the retrospective design introduced potential confounding variables and biases. Second, the natural course of HZ may lead to a gradual improvement in pain over time. Therefore, the study findings did not allow for a definitive inference that the US-guided intervention was the primary cause of pain improvement in the absence of a well-defined control group. Third, systemic absorption of corticosteroids may occur regardless of the injection approach, potentially contributing to pain relief in these patients. Finally, this study excluded patients with a high iliac crest, suggesting inherent technical limitations of the US-guided approach. Moving forward, a well-designed randomized controlled trial is necessary to validate our findings.

## Conclusion

In conclusion, US-assistant lumbar TFI using the retro-neural approach showed effective early relief for ZAP in the lower extremities. This approach is suggested as an alternative to the conventional FL-guided TFI because it provides significant clinical benefits, including an easier procedure with fewer needle attempts, shorter procedure duration, and decreased radiation exposure.

## Data Availability

The raw data supporting the conclusions of this article will be made available by the authors, without undue reservation.
